# Advancements in microalgal biomass conversion for rubber composite applications

**DOI:** 10.1038/s41598-024-82878-7

**Published:** 2025-01-04

**Authors:** Doaa S. Mahmoud, Salwa H. El-Sabbagh, Sayeda M. Abdo

**Affiliations:** 1 Polymers and Pigments Department, National Research Centre, Dokki, Giza, 12622 Egypt; 2 Hydrobiology Lab, Water Pollution Research Department, National Research Centre, Dokki, Giza, 12622 Egypt

**Keywords:** Microalgal biomass, Rubber composites, Curing behavior, Mechanical properties, Swelling properties, Characterization and analytical techniques, Chemistry, Engineering, Materials science, Nanoscience and technology, Physics, Environmental impact

## Abstract

Carbon black (CB) as rubber reinforcement has raised environmental concerns regarding this traditional petroleum-based filler, which is less susceptible to biodegradability. Although it has great reinforcing properties, the production technique is no longer sustainable, and its cost increases regularly. For these reasons, it is wise to look for sustainable replacement materials. Microalgal biomass (MB) has demonstrated great potential for use as biodegradable nano fillers in rubber composites. Microalgal has a high biomass productivity compared to traditional crops. They can produce a large amount of biomass per unit of land area, making them highly efficient in terms of resource utilization. In the present research, microalgal biomass was blended with CB at different concentrations for preparing two different kinds of rubber composites: Nitrile rubber Acrylonitrile-butadiene rubber (NBR) and styrene-butadiene rubber (SBR) are two common synthetic rubbers. In this study, the researchers investigated using microalgal biomass as filler in rubber composites. They assessed the filler-matrix interaction by evaluating the processability, mechanical characteristics, Payne effect, and swelling properties of the MB/CB-filled composites and compared them to CB-filled composites. The results show that rubber composites incorporating dual fillers (microalgal biomass and carbon black) had faster cure times, increased torque, and improved mechanical properties. The results prove biomass helps to minimize bulk quantities of CB and may be used as a partial replacement while still improving the mechanical properties. According to the study, microalgal biomass can successfully replace up to 50% of the CB filler. This will reduce petroleum dependence and possibly costs, depending on current petroleum prices.

**Supplementary Information:**

The online version contains supplementary material available at 10.1038/s41598-024-82878-7.

## Introduction

The demand for energy is increasing sharply due to the rapid growth of industrialization and urbanization, and the limited availability of conventional fossil fuels is not able to meet this demand. In addition, the uncontrolled consumption of fossil fuels and the careless use of natural resources have resulted in a number of serious issues like acid rain, global warming, desertification, acid rain, and air pollution, all of which offer a serious threat to the survival and advancement of humanity. To reduce the amount of dependency on fossil fuels, it is vital and necessary to look for sustainable, clean energy sources^1^. Global warming challenges are currently developing at an alarming rate and becoming more connected with others. Climate change is leading to an increase in the frequency and intensity of extreme weather events, such as heat waves, floods, and droughts. These events pose a growing threat to human security, as they can disrupt food and water supplies, damage infrastructure, and endanger lives. Meanwhile, the effects of global warming exacerbate problems including water scarcity, biodiversity loss, disease spread, and deteriorating soil. Patterns of political stability, economic progress, and human well-being are expected to be impacted by these shifts. Global warming is mostly caused by human activities, which have resulted in an increase in CO_**2**_, a significant greenhouse gas (GHG) that makes up to 60% of all greenhouse gases in the Earth’s atmosphere^2^.

Since CB has such high purity and superior reinforcing qualities, it has consistently been the tyre typical filler. CB possesses both chemical and catalytic action and may alter the vulcanization process of rubber. Although CB provides rubber composites with outstanding mechanical, thermal, physical, and barrier characteristics, it also has certain weaknesses. CB is produced through the incomplete combustion of petroleum-based materials. This process can release toxic gases and hazardous waste. Additionally, the International Agency for Research on Cancer (IARC) has classified CB as a potential carcinogen (group 2B), indicating it may be harmful to human health^3^. As a result, both the production and use of it harm the environment and cause several health issues. Therefore, in the rubber industry, it is essential to replace CB with an alternative, environmentally friendly, and renewable filler ingredient. Because of its affordability, sustainability, accessibility, and environmental friendliness, biomass is one of the most promising resources and has garnered interest worldwide^4,5^.

Microalgal biomass holds great promise for a variety of applications, but its application is restricted to lab environments. Industrial-level applications have not advanced due to the significant financial expenses related to large-scale implementations^6^. One of the best ways to address this issue is to cultivate microalgal using effluents, which has proven to be a practical solution for reducing process costs and producing microalgal biomass for a variety of applications^7^.

Microalgal biomass is a renewable biofiller naturally that can potentially replace CB to create rubber composites used in the tyre industry. As one of the most important aquatic plants, microalgal have been recognized as promising materials because they are inexpensive, renewable, and, most significantly, naturally biodegradable. Numerous studies show that microalgal can produce biomass from wastewater of all kinds, including municipal, industrial, agro-industrial, and livestock wastewaters, by completely removing nitrogen, phosphorus, and hazardous components^8,9^. Since the biomass of microalgal typically consists of proteins, lipids, carbohydrates, and colours, it is an excellent candidate for filler in polymers^10,11^. According to published research, algae can be employed in a variety of industrial processes, such as the synthesis of biobased polyols and bioplastics^12,13^. In accordance with its mechanical characteristics, acrylonitrile-butadiene rubber (NBR) is flame retardant, resistant to heat, oil, and hydrocarbon solvents. It is also impermeable to gases and volatile chemicals, water, and steam. While there is growing interest in using microalgal biomass for various applications, including rubber production, many studies are still preliminary. Comprehensive research is needed to understand the best practices for integrating microalgae into rubber materials. The most prevalent synthetic rubber is styrene-butadiene rubber (SBR) and acrylonitrile-butadiene rubber (NBR). A lot of research has been documented in the literature using different fillers to partially replace black.

Gobetti et al.^14^ investigate the potential of the primary steel industry byproduct, electric arc furnace (EAF) slag, as filler for a nitrile butadiene rubber matrix (NBR). Investigations were conducted into the impact of using slag in place of CB at equal filler volume % and hardness. The obtained results demonstrated that, in terms of hardness, viscosity, elongation at break, and crosslink kinetics, slag-filled NBRs are comparable to CB-filled NBRs.

Bordoloi et al.^15^ explored the utilization fly Ash (FA) encapsulated by environmentally friendly polysulphide produced from waste sulphur and spent canola oil. The compounds were compared to CB-only composites after a novel surface modified filler (SuMo FA) and stearic acid treated FA were used to partially replace CB. SuMo FA, promising waste-derived filler, is concluded to be able to partially replace carbon black in rubber composites without compromising thermal stability or mechanical performance. Caixin et al.^16^ have been shown that carbon black (CB) in NR can be partially replaced by nanocrystalline cellulose (NCC) obtained from waste cotton Additionally, they discovered that when 10% of CB was substituted with NCC, the mechanical, thermal, and biodegradability properties of NR/NCC composites were higher than those of NR/CB composites. Kazemi et al.^17^ Different contents of carbon black (CB) and cellulose fibers (before and after modification) were used as a hybrid filler system to investigate the possibility of CB substitution in NR composites. the results showed that the composite filled with 20 phr modified cellulose and 20 phr CB (50% replacement of CB) exhibited even better results than the composite filled with 40 phr of CB.

Peterson et al.^18^ Utilizing Pistachio Shell Biochar PSB reinforce natural rubber and reduce the total amount of CB in the composite, pistachio shell biochar would be suitable for partially replacing carbon black in applications like hoses, seals, belts, and gloves, thereby enabling a new application for this sustainable, agricultural waste product that will help reduce dependence on fossil fuels.

Bellinetto et al.^19^ utilized microalgal to potentially replace carbon black, a typical biofiller, in natural rubber compositions through renewable sources. ***Spirulina*** was found to perform similarly to N990 by improving rubber tensile strength and elasticity up to 50 phr, but it also decreased rubber thermal resistance and fracture resistance. Rajan et al.^20^ look into how the final properties of the compound changes when graphene is partially substituted for carbon black. An efficient interaction between the elastomer and the modified graphene was evidenced by the increased mechanical properties detected with a partial replacement of carbon black with a modest amount of graphene.

Torres et al.^21^ used poly(butylene adipate-co-terephthalate) (PBAT) to fabricate biocomposites from residual microalgae biomass (RMB). According to the experiments, RMB can be used to create biocomposites with PBAT; 20% RMB provides the best extrusion findings. The best outcome was obtained with 7.5 phr of urea and 30% glycerol. The potential of high-proteinaceous microalgae in the production of bioplastics was investigated by Zeller et al.^22^. They discovered that whereas Spirulina microalgae demonstrated superior mix performance, Chlorella demonstrated superior bioplastic behavior. Mohamed et al.^23^ Applied microalgae to produce the raw materials for more efficient bioplastic production is an important aspect. They demonstrate that microalgal strains have the most potential for creating bioplastics. Ciapponi et al.^24^ use microalgal biomass as a filler, biobased plasticisers (glycerol, octanoic acid, and 1,4-butanediol), and wheat gluten to produce new bioplastic compounds. They observed that a more effective dispersive mixing of algae in the bioplastic matrix could further enhance mechanical performance.

CB is commonly used filler in rubber composites, but it is derived from the incomplete combustion of petroleum products and has been classified as a potential carcinogen. In an effort to find more sustainable alternatives, researchers have explored the use of biochar derived from coppiced hardwoods as a partial replacement for carbon black in rubber blends containing natural and butadiene rubber. The findings suggest that the biochar-containing composites exhibited increased elongation and toughness compared to the traditional CB-filled samples, while maintaining similar levels of tensile strength^11^.

The current study attempts to reduce the environmental problems by partially replacing CB and using a readily available renewable biofiller at the same time. by using the algal biomass collected from a high rate algal pond designed for wastewater treatment to create a value-added product. Many analytical methods, including FTIR, XRD, SEM, TEM, and TGA, are proposed to be used to characterize microalgal biomass that was produced. An investigation is being proposed on the different mechanical, physical, barrier, thermal, and swelling properties of synthetic rubber with microalgal and CB.

## Experimental procedures

### Materials

Acrylonitrile-butadiene rubber (acrylonitrile content: 32%) provided from Bayer AG, Germany and Styrene-butadiene rubber (styrene content: 23%) purchased from Transport and Engineering Company (TRENCO) in Alexandria. Carbon black (N-330) was obtained from OCI Co. Ltd. Commercial compounds like Zinc oxide (ZnO) and stearic acid (St Ac) activators were incorporated as received, along with polyethylene glycol (PEG) from Sigma-Aldrich. German company Aldrich supplied commercial-grade N-cyclohexyl-2-benzothiazole sulfenamide (CBS), the elemental sulfur (vulcanizing agent), and polymerized 2,2,4-trimethyl1,2-dihydroquinoline (TMQ) (antioxidant).

## Preparation

### Microalgal strain preparation

#### Growing algal biomass grown in HRAP

Construction of a pilot scale is paving the route to upgrade the stabilization ponds on large scale (S1). At the Zenin Wastewater Treatment Plant in Giza, Egypt, which is situated at 30° 1’57.91"N/31° 10’53.53"E, average daily temperature variation in the selected land region for this pilot facilities are 10 °C, while seasonal temperature range varies between 15 and 40 °C.

#### Microalgal l performance and identification for algal community structure

The algal identification process involved the following steps:

Collecting sub-samples from different depths within the High rate algal pond. Mixing the sub-samples to form a single, composite sample then allowing the composite sample to settle overnight. Examining the settled sample using Sedgwick-Rafter counting cells, in accordance with the identification keys for freshwater algae^25^. Microscopic examination (using an Olympus X3 microscope, Olympus Corporation, Tokyo, Japan) to identify the algal community at the species level.

**Fig. 1:**
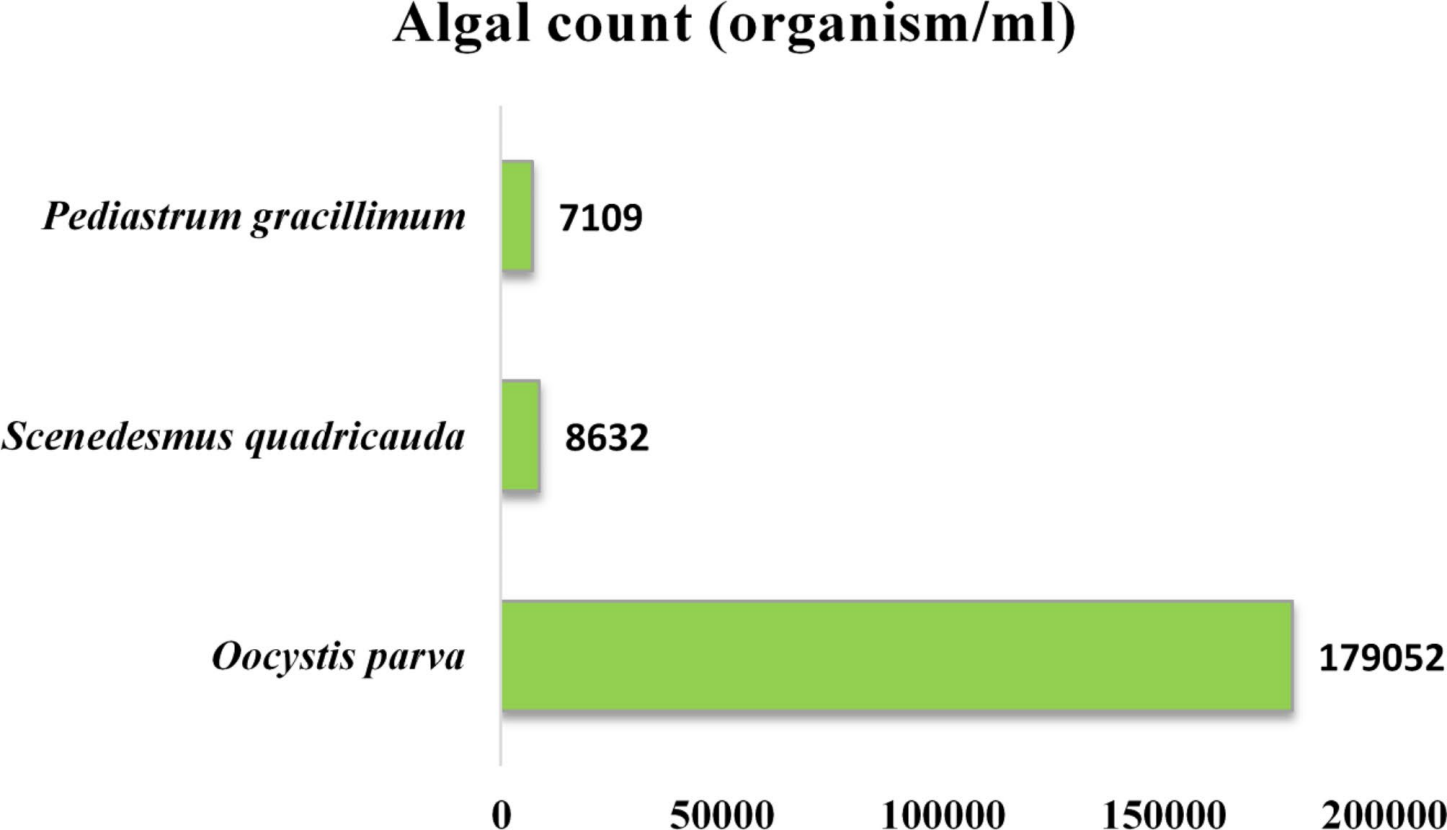
Algal community structure of microalgal biomass collected from High Rate Algal pond.

The key aspects of the algal identification procedure were the collection of sub-samples from various depths, the formation of a composite sample, the sedimentation process, the use of Sedgwick-Rafter cells, and the final microscopic examination to determine the species-level composition of the algal community (as shown in Fig. 1).

#### Harvesting and drying algal biomass

The algal biomass (MB) was collected and then allowed to settle in order to lower the amount of water. After the settling process, the excess water was discarded, leaving behind the concentrated algal biomass. The settled algal biomass was then spread out onto trays and placed inside a solar dryer. The solar dryer was set up in a field environment, where the temperature consistently exceeded 40 °C. The use of the solar dryer allowed the algal biomass to undergo drying, leveraging the high ambient temperatures in the field location. This solar drying process helped to further remove the remaining moisture from the algal biomass, resulting in a dried, concentrated algal material that could be used for subsequent analysis or applications.

#### Grinding dried algal biomass in a Planetary Ball Mill

For the purpose of grinding the algal biomass, we utilized a planetary ball mill (Retsch GmbH, Haan, Germany) that came equipped with a grinding jar and 1 mm beads made from yttrium-stabilized zirconium oxide. To ensure optimal performance and prevent overheating, we paused the grinding process every 10 min to allow the jar to cool. Following the grinding procedure, we proceeded to separate the beads from the algal biomass using a sieve.

## Rubber based composites preparation

**Fig. 2:**
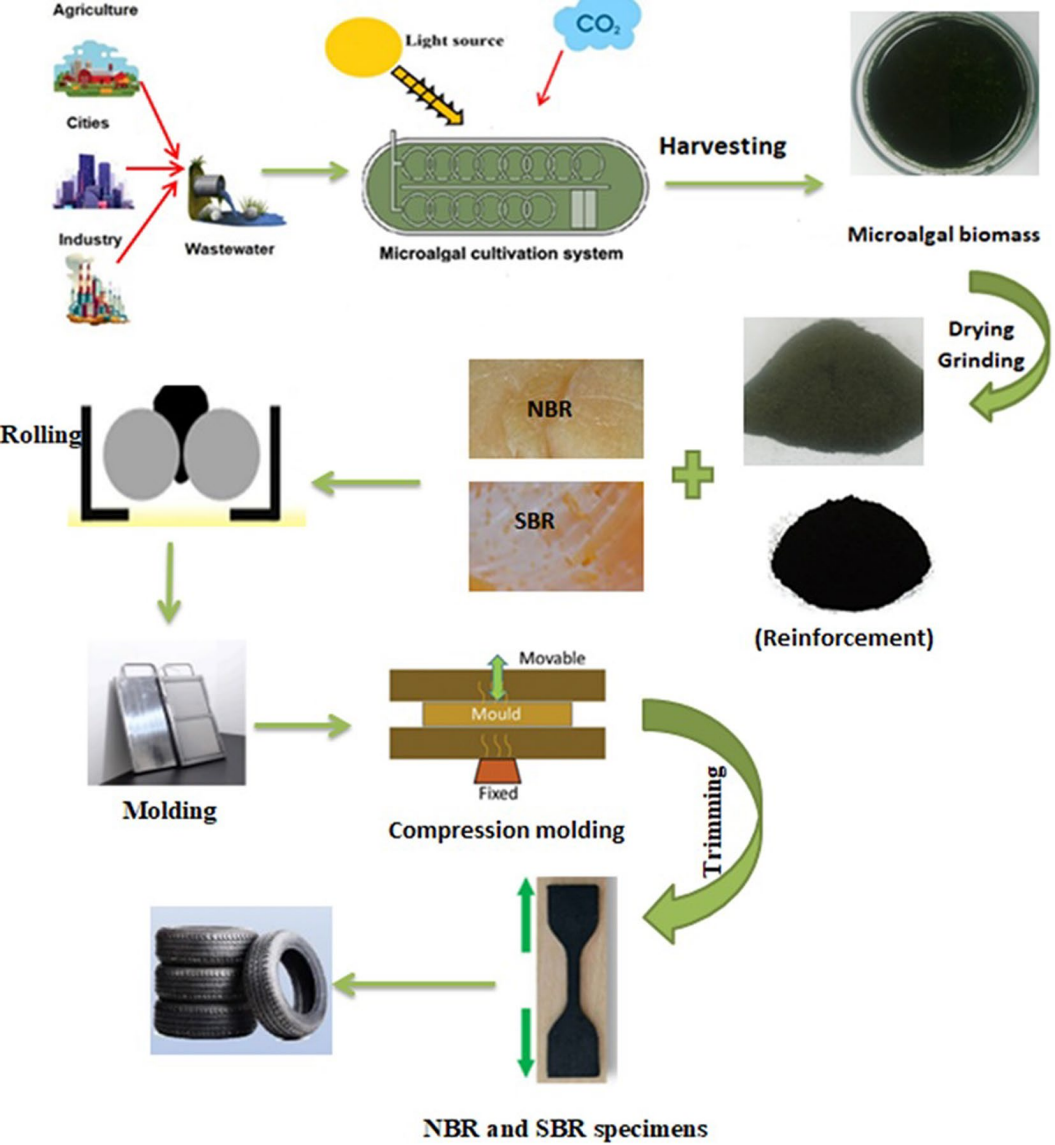
Schematic for the fabrication process of rubber-based composite via the two-roll mill method.

Table 1 shows different formulations of samples prepared for this work. On the basis of this table, NBR and SBR were selected as the matrix materials. There were two steps involved in mixing. First stage included using two roll mills at room temperature to combine rubbers and all additives other than sulfur and accelerators. After masticating NBR and SBR for two minutes, co-activator SA and activator ZnO were added until the mixture was homogenous. After another two minutes of mixing, the fillers with coupling agent (PEG)^26,27^ and antioxidant (CBS) were added, and mixing proceeded for an additional five minutes. The biomass/CB composites were formed by mixing rubber and biomass/CB (mass ratio (1:1)) in different ratios between 0 and 60 parts of MB/CB per hundred rubbers (phr). Reference unfilled NBR and SBR samples were also prepared for comparison purposes. The anti-degrading accelerator (TMQ) and vulcanization agent S were added at the end of mixing to initiate the curing process (as shown in Fig. 2). After achieving uniform mixing and homogenization, the resulting composites were cured at 152 °C for T_**c90**_ as determined by a rheometer under pressure. The rubber composites were sheeted out with a thickness of about 4 mm and maintained at room temperature for a full day prior to performing the compound characterization investigations.

**Table 1:** Composition of rubber composites.

Materials	Samples code											
	NB-0	NB-1	NB-2	NB-3	NB-4	NB-5	SB-0	SB-1	SB-2	SB-3	SB-4	SB-5
NBR	100	100	100	100	100	100	–	–	–	–	–	–
SBR	–	-	–	–	–		100	100	100	100	100	100
ZnO	5	5	5	5	5	5	5	5	5	5	5	5
St. acid	2	2	2	2	2	2	2	2	2	2	2	2
Microalgae biomass	–	–	7.5	15	22.5	30	–	–	7.5	15	22.5	30
CB	–	15	7.5	15	22.5	30	15	15	7.5	15	22.5	30
PEG	–	1	1	1	1	1	1	1	1	1	1	1
CBS	0.8	0.8	0.8	0.8	0.8	0.8	0.8	0.8	0.8	0.8	0.8	0.8
TMQ	1	1	1	1	1	1	1	1	1	1	1	1
S	2.5	2.5	2.5	2.5	2.5	2.5	2.5	2.5	2.5	2.5	2.5	2.5

## Characterization

Using a JASCO FTIR-6000 E Fourier transform infrared spectrometer (Japan), FT-IR spectra were acquired through mixing with discs of potassium bromide (KBr) and operating in the absorption mode between the 4,000–400 cm^–1^ wave number range. The Model ATR PRO450-S, Single Reflection Measuring Attachment was used to gather the spectra at a resolution of 4 cm^− 1^. Rubber composites were subjected to energy-dispersive X-ray analysis (EDAX) and scanning electron microscopy (SEM) using Quanta FEG 250, which was connected to the EDAX unit. EDAX was employed to identify elements on any compound’s surface. Utilizing a micro analyzer electron probe, the investigated substance was evaluated using a JEOL JEM 2100 transmission electron microscope (TEM) from Japan. Utilizing Perkin Elmer analyzer equipment from the USA, the thermal gravimetric analysis (TGA) test was performed. Using a heating rate of 10 ºC/min and a nitrogen air flow of 50 ml/min, sample weights ranging from 13 to 25 mg were scanned between 50 and 1000 ºC. Particle size measurement used to estimate the samples’ zeta potential, size distribution, and average diameter, a particle size analyzer (Nano-ZS, Malvern Instruments Ltd., UK) was used. The sample had been sonicated for 10–20 min prior to the assessment to measure the zeta potential and size distribution.

Cytotoxicity’s effect on human cell lines: The vitality of the cells was assessed by measuring the mitochondrial-dependent conversion of yellow MTT (3-(4,5-dimethylthiazol-2-yl)-2,5-diphenyl tetrazolium bromide) to purple formazan.

Procedure:

Every procedure that followed was carried out in a sterile environment with a Laminar flow biosafety cabinet Class II A2 (made by Labconco). In a CO_**2**_ incubator (Sartorius stedium, biotech) with 5% CO2, 1% L-glutamine, 5% fetal bovine serum, and 1% antibiotic-antimycotic combination (10,000U/ml Potassium Penicillin, 10,000 µg/ml Streptomycin Sulfate, and 25 µg/ml Amphotericin B) at 37 ºC, the cells were suspended. Following a 10-day batch culture, cells were seeded at a density of 10 × 103 cells/well in newly prepared growth medium in 96-well plastic plates. After that, the plates were maintained at 37 °C with 5% CO2 for a whole day, either with no drugs (by way of a negative reference) or with different drug concentrations to produce a final concentration of (1000, 500, 250, 125, 62.5, 31.25, 15.625 µg /ml). After the medium was withdrawn after 48 h of incubation, 20 µg of MTT salt (2.5 µg/ml) was added, and the mixture was then incubated for four more hours at 37 ºC using 5% CO_**2**_. 200µL of 10% Sodium dodecyl sulphate (SDS) in 0.01 M HCL has been introduced to every well, and the mixture stored overnight at 37 ºC to inhibit the reaction and dissolution of the produced crystals. A well-known cytotoxic natural element that is 100% lethal under the identical conditions was utilized as a positive control, consisting of 100 µg/ml^28,29^. Next, utilizing a microplate multi-well reader (Bio-Rad Laboratories Inc., model 3350, Hercules, California, USA) and a reference wavelength of 620 nm, the absorbance was measured at 595 nm.

1 $$Viability=\left( {\frac{{absorbance\;of\;drug}}{{absorbance\;of\;control}}~ \times ~100} \right)$$

2 $$cytotoxicity=100 - viability$$

Employing a Monsanto oscillating disc rheometer-100 (ASTM D 2084-07, 2007a), the rheometric properties of NBR and SBR compounds M_**L**_ (minimum torque), M_**H**_ (maximum torque), T_**c90**_ (optimum cure time), T_**S2**_ (scorch time), and cure rate index were explored. In a hydraulic press, the compounded rubber was vulcanized at 4 MPa of pressure and 152 ± 1 ºC for NBR and SBR. Following ASTM D412-06a (2013), dumbbell-shaped samples were cut from vulcanized rubber sheets and put through tensile testing on a Zwick testing equipment. For each rubber composite sample, five mechanical property-related variables were measured: tensile strength (*σ*R), tensile stress at 100, 200, and 300% strain and strain at break (*ε*R). Data analysis was conducted using the average results obtained from testing at least five replicate specimens.

## Results and discussion

### Biomass analysis

#### Algal community structure

**Fig. 3:**
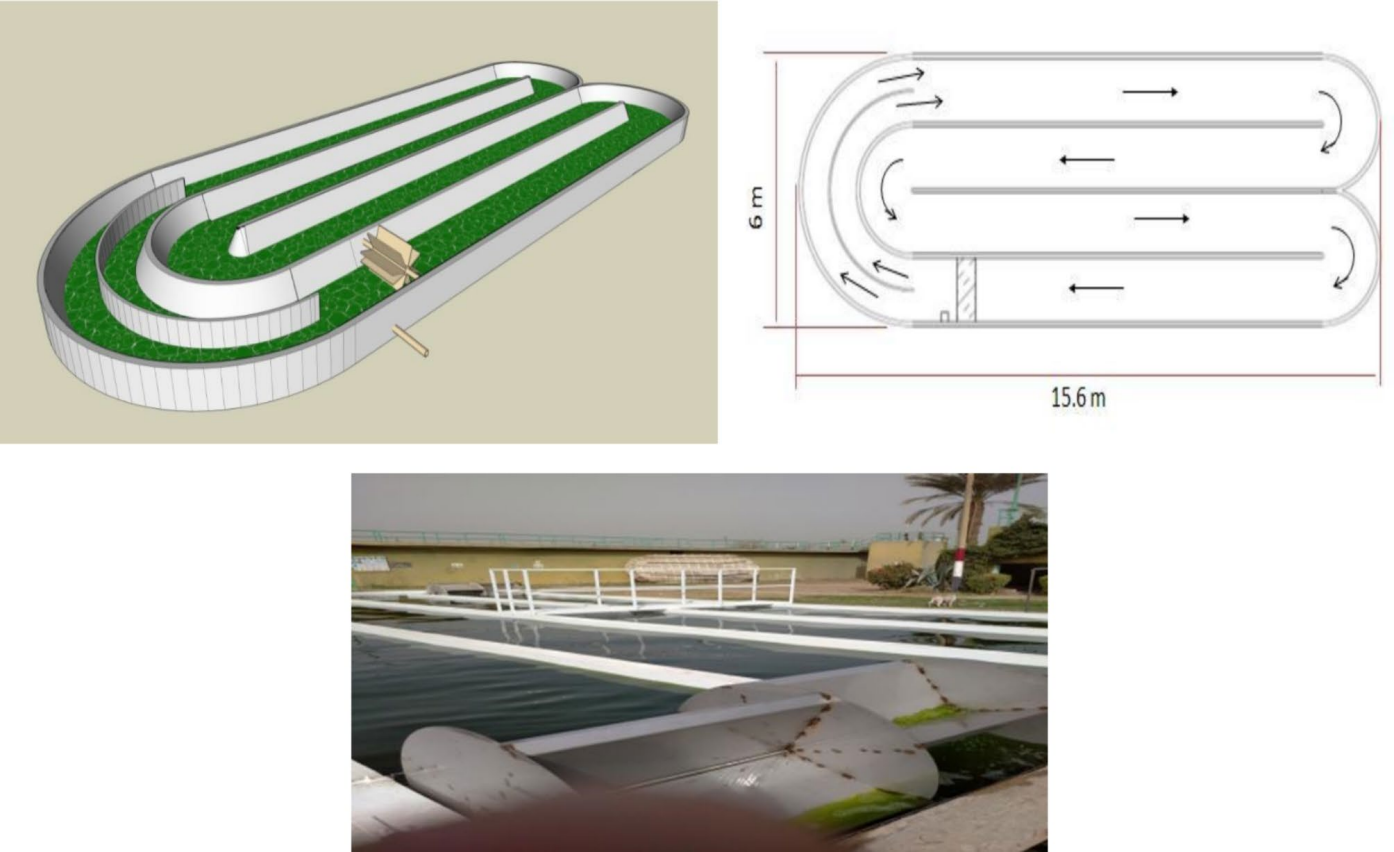
High Rate algal pond at Zenin wastewater treatment plant.

The algal community structure identified in samples collected from a high rate algal pond was dominated by *Oocystis parva*, which belongs to the Chlorophyta (green microalgal group) as shown in Fig. 3. In addition to *Oocystis parva*, the presence of *Scenedesmus quadricauda* and *Pediastrum gracillimum* was also observed (S2). These green microalgal species are commonly found in freshwater environments, including wastewater treatment systems. It is known for its ability to form distinctive colony structures, which can contribute to the overall diversity and stability of the microalgal community in the treatment process. The presence of *Oocystis parva*, *Scenedesmus quadricauda*, and *Pediastrum gracillimum* in wastewater treatment systems indicates the ability of the system to support the growth of these nutrient-removing microalgal species^30–32^. The quantitative representation of these three microalgal strains is revealed in Fig. 3. High levels of organic matter and nutrients in wastewater can lead to eutrophication if not adequately treated before entering natural water bodies. Thus, municipal wastewater (MWW) treatment is crucial for preventing eutrophication, protecting the environment, and reducing waterborne disease risks. While current large-scale treatment facilities are effective for many pollutants, they often fail to remove specific contaminants like nitrogen, phosphorus, and micropollutants. This gap has sparked interest in alternative methods, particularly biological processes using microalgae, which offer a sustainable and efficient approach to nutrient recovery and wastewater treatment^33–36^. The recovery of microalgae biomass after treatment is a crucial step in wastewater management, providing potential applications across various fields^32^. While the potential for using microalgae in rubber applications is promising, the field requires further exploration and development before it can be considered mature or commercially viable.

### FTIR spectra

**Fig. 4:**
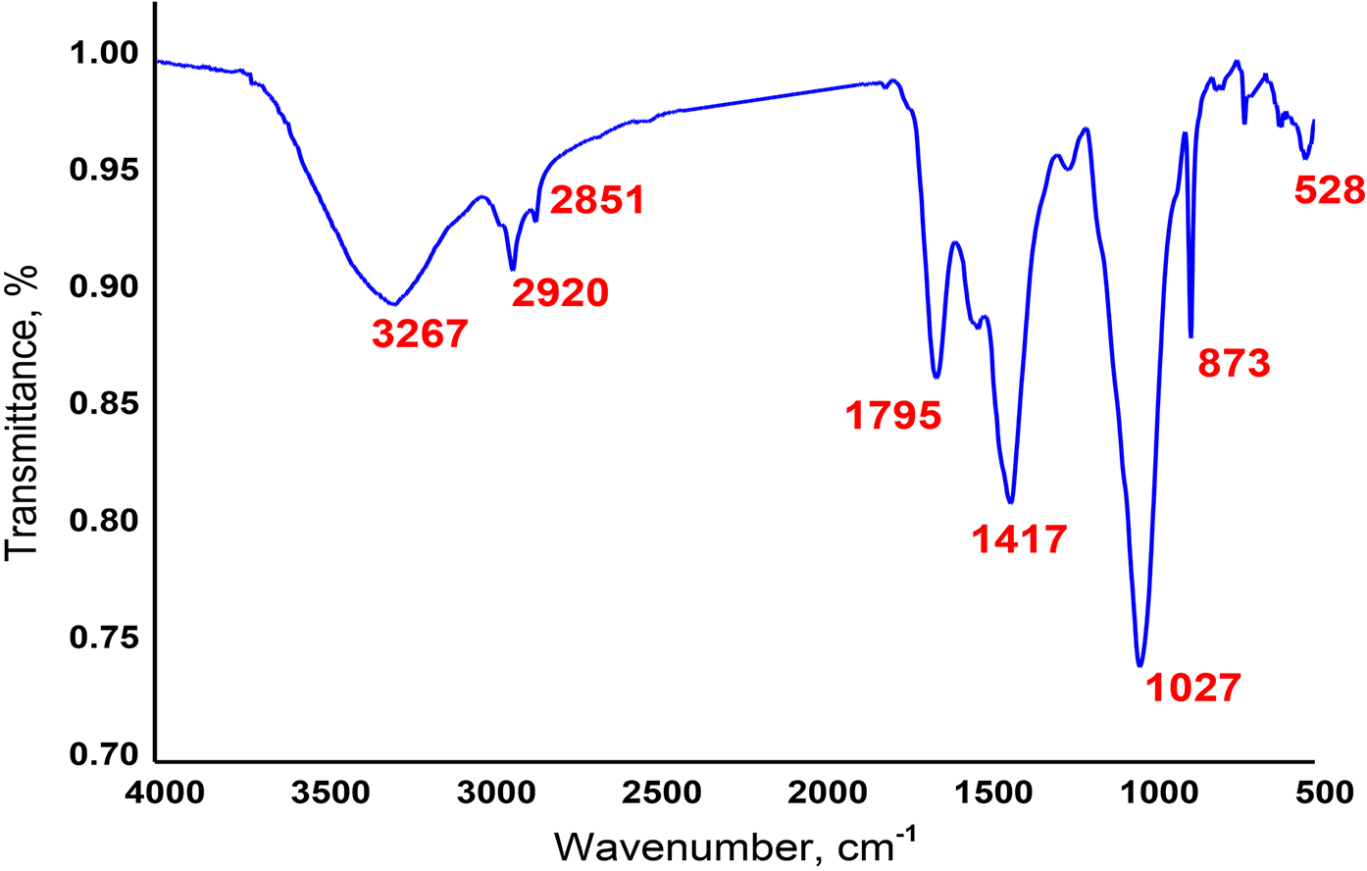
FTIR spectra of microalgal biomass.

The position, breadth, and intensity of infrared light absorbed can be used to assess the constitution and molecular functional groups. Figure 4 exhibits the MB FTIR data and reveals the presence of organic compound groups. The absorption spectra displayed discrete bands that, over the wave number range of 4000 –500 cm^− 1^, corresponded to several biomolecules, including proteins, lipids, and carbohydrates. The bands observed between 3500 and 2800 cm^− 1^ are asymmetric and symmetric stretches of hydrocarbons from lipids, specifically CH_**3**_ and CH_**2**_. Carbonyl stretching from fatty acid esters (triacylglycerides) was responsible for the band observed between 1700 and 1500 cm^− 1^. The bands at 3277 cm^− 1^ reveal the presence of a hydroxyl group. This result is in agreement with^37^. The band at 1027 cm^− 1^ confirms the presence of C-O-C polysaccharides from carbohydrates. The aliphatic groups verify a noteworthy increase in the quantity of hydrocarbons in the aliphatic group region at 528 cm^− 1^. The FTIR analysis of the MB revealed the presence of alcohol, lipid, and carboxyl functional groups, indicating the presence of hydrocarbons. Based on these findings, microalgal can be a promising sustainable raw material for various applications^38,39^. By analyzing the spectra, researchers can assess how microalgae might behave as raw materials in different products (e.g., biofuels, bioplastics, rubber composites). For instance, the presence of certain functional groups may indicate good compatibility with other materials or desirable properties like elasticity or durability^40^.

### SEM-EDAX and TEM analyses

**Fig. 5:**
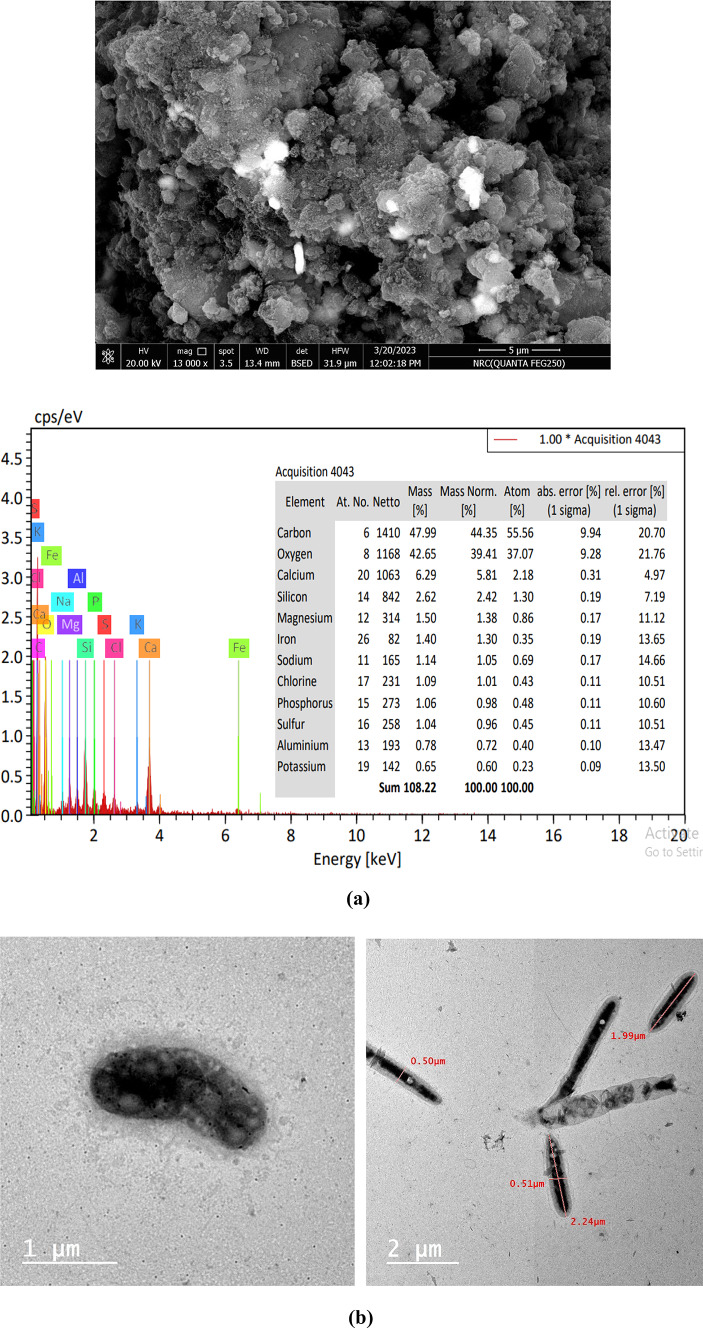
(**a**) SEM-EDEX of microalgal biomass. (**b**) TEM of microalgal biomass.

SEM analysis of the MB was done to assess the surface appearance and shape during the milling process. Microalgal has a jagged surface and normal-shaped particles, as illustrated in Fig. 5a. The majority of the MB particles are irregular in form; some are spherical and have a rough surface, whereas the other aggregates have been unevenly formed. Additionally, the EDAX spectrum of the biomass sample is shown in Fig. 5a, allowing information regarding the surface composition of the biomass to be gathered. The weight and atomic percentage data showed that MB had a high concentration of the three primary components of microalgal, C, O, and N. The biomass was enriched with different inorganic elements.

The TEM micrographs of MB show distinct morphologies with characteristic rough surfaces, together with the outline of a regular cell wall, plasmalemma adjacent to the cell wall, other organelles within the cell, a prominent nucleus, and uniformly distributed vacuoles throughout the cytoplasm (Fig. 5b).

### Particle-size measurement

**Fig. 6:**
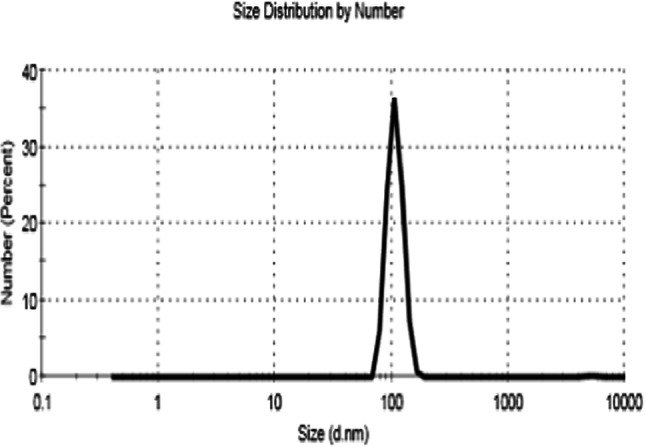
Particle-size distribution of biomass powder.

A particle-size analyzer was used to characterize the MB particles, as shown in Fig. 6. According to the particle-size analysis results, the majority of the MB particles were between 99 and 107 nm in size, with a particle size range of 0.5 to 5277 nm. Figure 6 illustrates how biomass was collected, dried, and pulverised into minute particles. Following drying, the average particle size of algal biomass was determined using dynamic light scattering. It was discovered that the algal biomass was prepared in modest quantities due to the possible effect of the ball milling mechanism. The particles predominantly measure 107 nm in size, “The powder has a polydispersity index (PdI) of 0.2450, suggesting that it is homogeneous.”

### Cytotoxicity evaluation

An assessment was conducted between the MB and the human cancer cell line HePG 2 (Human hepatocellular carcinoma cell line). Sample concentrations between 20.000 and 312.5 µg/mL can be measured with the MTT test. Human tumor cell lines’ estimated cytotoxicity was verified using the MTT assay’s IC50 values and percentage of viable cells. 48 h at 37 °C and 5% CO_**2**_ were employed to test the biomass at dosages ranging from 1000 to 15.63 µg/mL. 50% of cells die within 48 h due to the sample’s lethal concentration (IC 50) of 1082 µg/mL. This proves there was no cellular harm from the MB^41^.

### TGA result

**Fig. 7:**
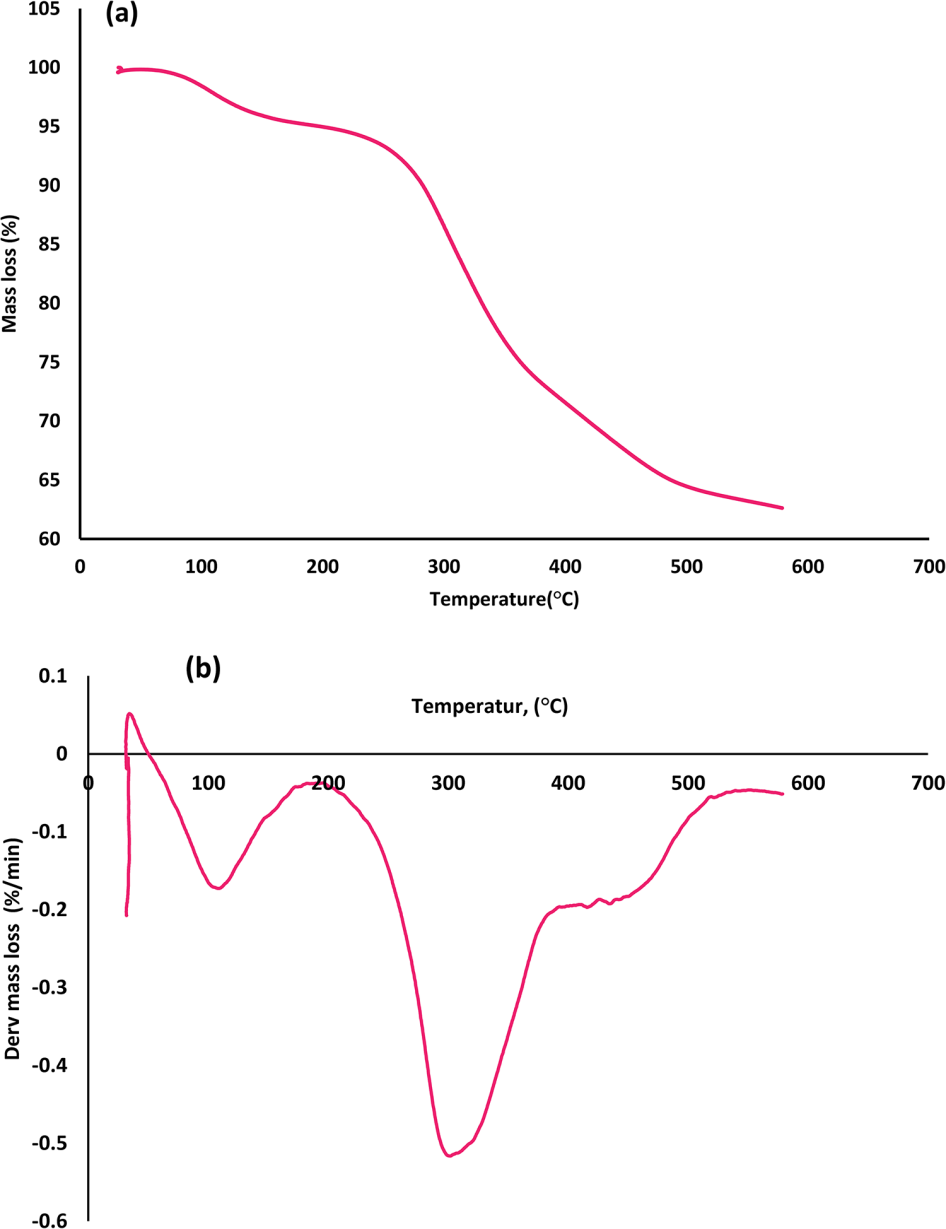
TGA (**a**) and DTG (**b**) curves of microalgal biomass.

The weight loss curves derived from the TGA curve for the MB are displayed in Fig. 7. Three decomposition events are revealed by the TGA for the microalgal, including one at < 110 °C that corresponds to the algal biomass’s dehydration from external water, 110–480 °C related to devolatilization and > 480 °C, which denotes the solid residue’s slow degradation from the last stage. These findings allow us to see that the main decomposition stage, that takes place between 110 and 480 °C, is actually a very complicated process^39^. The degradation of proteins and carbohydrates in this stage leads to a more notable mass loss Moreover, a smaller peak and a shoulder were caused by the thermal degradation of lipids since their pyrolytic temperature was higher than that of proteins and carbohydrates. In contrast, the MB exhibited a 71% mass loss in the temperature range of 110 to 480 °C. The third step corresponds to the degradation of solid residue, which denotes the non-volatile substance that remains in the TGA pan as the temperature rises. At a temperature of 480–567 °C, the biomass showed a 9% mass loss^39,42^. According to the DTG curve (Fig. 7b), MB had a higher peak decomposition temperature of 303.2 °C. The peak that was detected suggests that the biomass contains chemical components that possess high thermal stabilities.

### Composites characterization

#### FT‑IR spectroscopy of the prepared composites

**Fig. 8:**
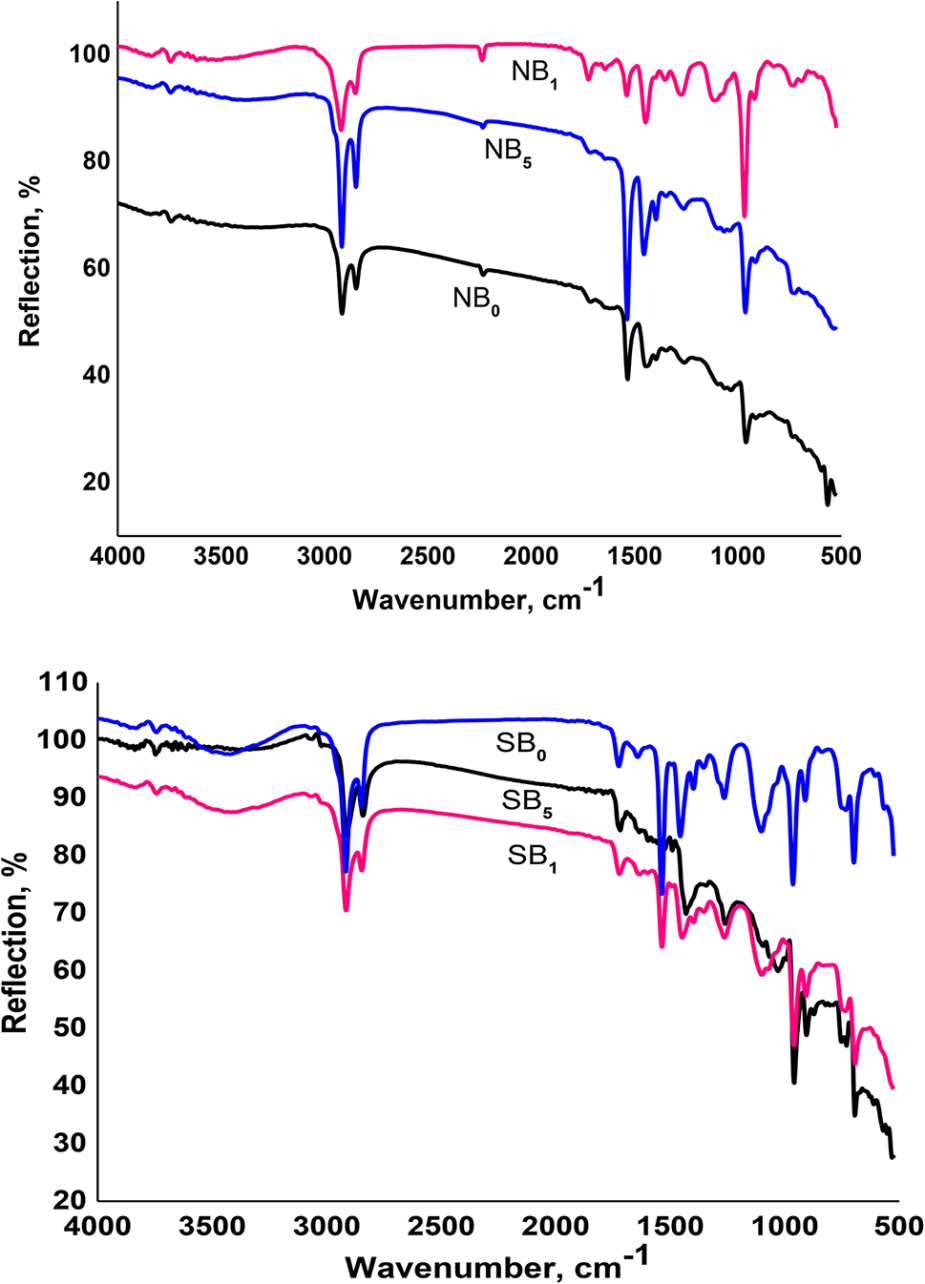
FTIR spectra of NBR and SBR based matrix and their composites containing carbon black (CB) and microalgea biomass (MB).

To validate the inclusion of CB and MB, FT-IR spectroscopy of NBR vulcanizates was performed. Figure 8a displays the NBR, NBR/CB, and NBR/CB/MB FT-IR spectra. Asymmetric and symmetric C–H groups of NBR are responsible for the stretching vibrations that are detected at 2959 cm^− 1^, 2851 cm^− 1^, and 1446 cm^− 1^, respectively, in all of the spectra that are observed. Furthermore, the stretching vibration of NBR’s C=C– and = C–H is responsible for the strong band at 1626 cm^− 1^. Alkyl C–N stretching vibrations of acrylonitrile are responsible for the band at 2230 cm^− 1^, which is consistent across all spectra. Figure 8b displays the FTIR spectra of the SBR samples. The C-H stretching of SBR molecular chains was reflected by the notable peaks observed at 2846 cm^− 1^ and 2913 cm^− 1^ in the pure SBR (SB_0_) sample. Concurrently, a significant peak detected at 1712 cm^− 1^ was attributed to the vibration of the C = C double bonds inside the SBR aromatic ring. Furthermore, the SBR backbone structure’s C-C bending was attributed to the peaks at 1259 cm^− 1^, 1444 cm^− 1^, and 1533 cm^− 1^. Furthermore, the 960 cm^− 1^ peaks were connected to the = C-H bending vibration of SBR, whereas the 734 cm^− 1^ peaks were ascribed to the CH_**2**_ rocking vibration^43^. MB species in both NBR and SBR composite are shown in Fig. 8a and b, which demonstrate the existence of organic chemical groups. The absorption spectra showed distinct bands of absorption in the 4000–500 cm^− 1^ wave number range that correlated to various biomolecules, such as proteins, lipids, and carbohydrates. The bands seen at 2850 cm^− 1^ include asymmetric and symmetrical stretches of hydrocarbons from lipids that are CH3 and CH_**2**_. Increasing the amount of MB in both NBR and SBR composites increases the band’s intensity. The band at 1620 cm^− 1^ was caused by carbonyl stretches seen in fatty acid esters (triacylglycerides). Carbohydrate (C–O–C vibrate) polysaccharides are visible in the bands at 964 cm^− 1^.

#### Microscopic characterization

**Fig. 9:**
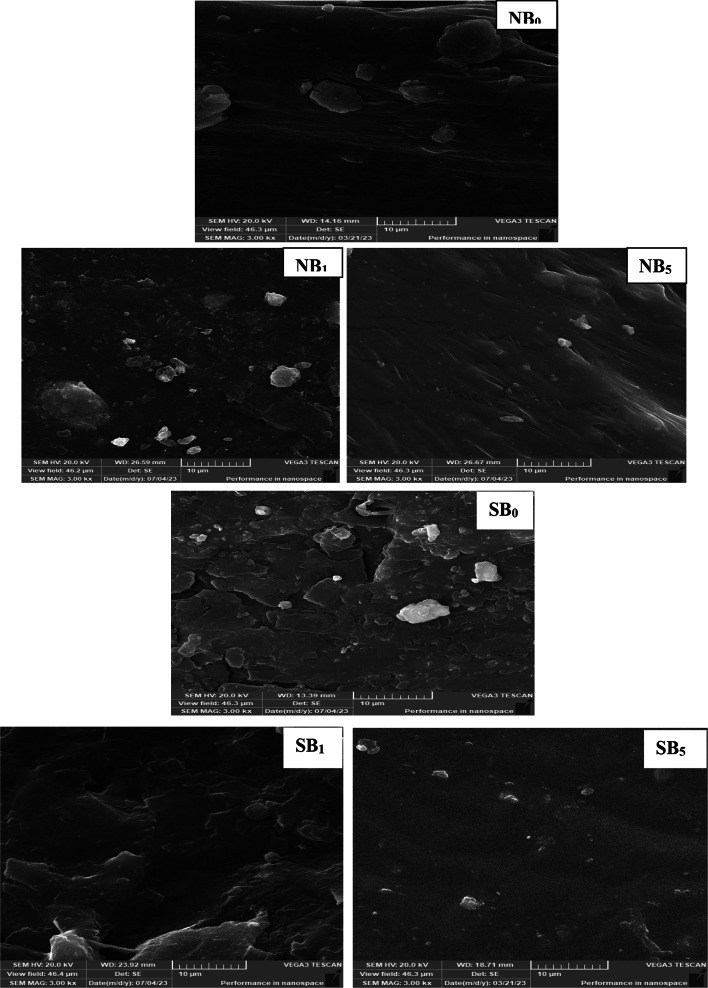
SEM images of NBR and SBR based matrix and their composites containing carbon black (CB) and microalgea biomass (MB).

The fracture surface of elastomeric composite materials and the state of dispersion were assessed. Figure 9 depicts SEM micrographs of the selected NBR and SBR composites containing CB (15phr) and CB/MB (30/30phr). The image of control sample (NB0), denoted as 0, has a smooth surface and exhibits an elastomer-typical fracturing mechanism. The control sample (SB0) exhibits matrix deformation on its fracture surface, and tiny agglomerates are visible. These agglomerates can be explained by ingredient aggregates that form during the composites’ processing. The topography of the surface becomes more uneven and rough when intercalated CB is employed as filler in NBR and SBR at the level of 15 phr^44^. The ridges, branches, creases, and undulations are seen, indicate effective stress transfer in NB_**1**_ and SB_**1**_. Figure 9. illustrates the morphological changes that occur when MB partially replaces CB. The biomass is finely distributed throughout the NBR matrix, and there is no evidence of particle detachment, suggesting that the filler improves rubber adherence. An efficient crosslinking process is suggested by a smooth matrix surface. From the SEM pictures, it can be concluded that when MB is added, the rubber matrix and filler have excellent compatibility (there are no gaps on the rubber-biomass boundary); nevertheless, furthermore there is an affinity for the particles of MB make aggregates due to significant interactions among the filler particles. The SBR composites also showed a similar type of behavior but the surfaces particles more smooth and flat. Furthermore, it can be observed that the partial substitution of CB resulted in a more flexible break in the cryofracture, indicating a superior mechanical property due to the chains’ restricted movement^45^. MB particle dispersion in SBR composites is inferior to that in NBR. This can be explained by the fact that hydrophilic fillers never have sufficient interactions to create fine dispersion or good adhesion with nonpolar polymers like SBR.

#### Rheometric characteristics

**Fig. 10:**
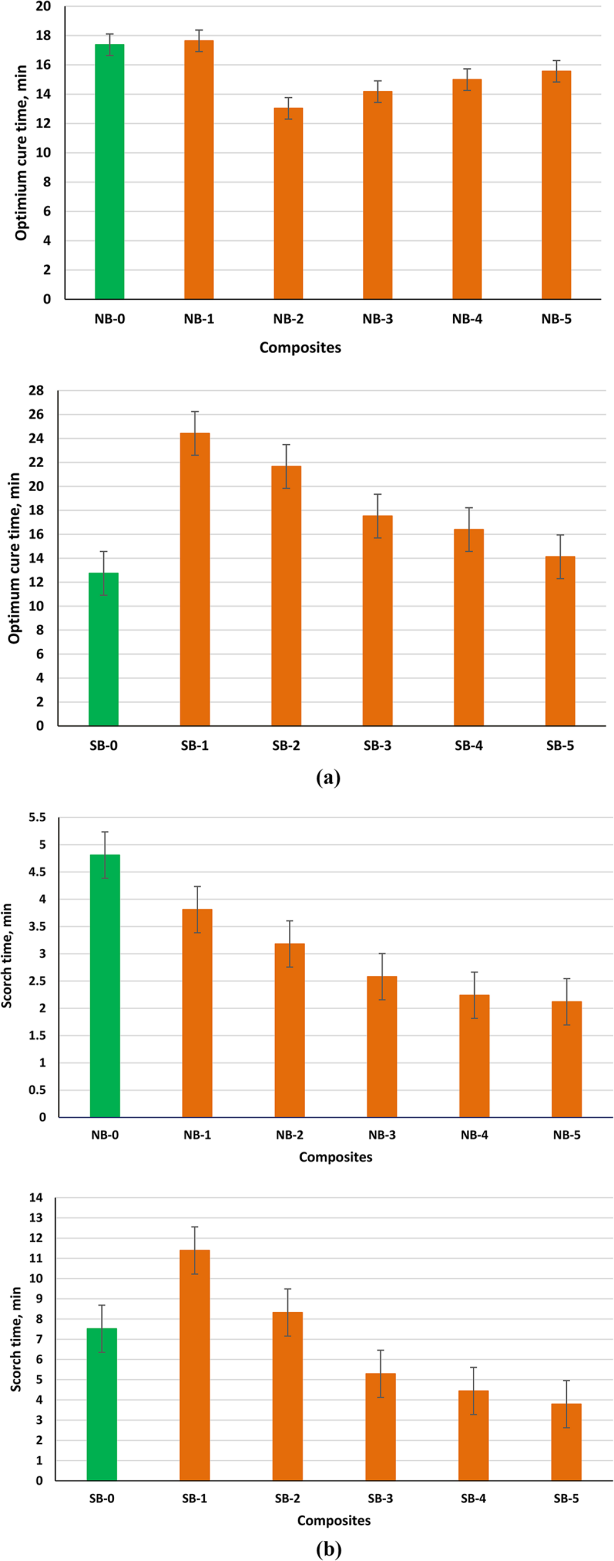
(**a**) Optimum Cure time for NBR and SBR composites with MB/CB in different proportions. (**b**) Scorch time for NBR and SBR composites with MB/CB in different proportions.

Rheometry results for the composites under investigation are given in Fig. 10; Table 2. The cure time (t_**C90**_) measurements reveal that increasing the amount of MB in the NBR and SBR composites at varying ratios to partially substitute CB accelerated the cure process. T_**C90**_ is crucial in evaluating the industrial operation window and represents the amount of time necessary to obtain a 90% complete state of cure. On the other hand, the material needs to vulcanize quickly in order to shorten cycle times, promote production, and minimize costs^46^.

**Table 2:** Rheometric parameters of rubber compared composites.

	Samples code											
	NB-0	NB-1	NB-2	NB-3	NB-4	NB-5	SB-0	SB-1	SB-2	SB-3	SB-4	SB-5
MH, dNm	9.13	13.5	11.94	14.12	16.81	17.97	12.99	10.5	10.25	12.1	11.53	11.44
ML, dNm	0.45	0.5	0.47	0.52	0.72	0.85	0.65	0.8	0.69	0.97	1.18	1.56
DM, dNm	8.68	13	11.47	13.60	16.09	17.12	12.34	9.7	9.56	11.13	10.35	9.88
CRI, %	7.962	7.23	10.15	8.95	7.84	7.44	9.16	7.67	7.48	8.18	8.36	9.68

In contrast to CB/rubber composites, NBR and SBR filled with the hybrid filler (biomass/CB) have shorter curing times. Other researchers have also corroborated this result^19^. This makes sense in considering the fact that MB have proteins with -OH groups that may combine with sulfur-forming polysulphide intermediates and accelerators to quick up the vulcanization process^47^. Figure 10a shows that the T_**C90**_ of NBR composites significantly increased with an increase in the mass ratio of microalgal. The functionalized MB includes a high amount of surface-located functional groups that interface with accelerators. Furthermore, the hybrid filler networks restricted the distribution of curing agents within the rubber matrix. In all ratios, the t_**S2**_ decreased with biomass concentration, indicating less clumping, improved dispersion and filler-filler interaction, and a faster cure (Fig. 10b). The PEG and MB interacted and reacted chemically, producing different types of cross-links. Our results support previous studies^27^.The type and amount of filler affect the minimum torque (M_**L**_) that indicates the processing features of rubber mixes and the stock viscosity of the uncured composites. According to the data obtained (Table 2), it was found that NBR and SBR matrices with greater biomass/CB mass ratios were also characterized by higher M_**L**_ values, which increased the aggregation of the fillers dispersed throughout the rubber matrix^45^. Sample NB-2 had the lowest reported M_**L**_ value, which may have contributed to the composites’ good mechanical properties and effective dispersion of the MB within NBR matrix^48^. The utilization of biomass/CB as a hybrid filler enhances the M_**H**_ values compared to composites without filler and, also most evident in NBR composites with higher MB concentrations^49^. The surface area of the MB particles is thought to be responsible for the increase in M_**H**_ because it supports more biomass/matrix interaction and increases material stiffness. Other researchers have confirmed this impact as well^50^. In general, the crosslink density is proportional to the differential torque, or ΔM, meaning the difference between M_**H**_ and M_L_. The ΔM SBR composites have been found to increase with an increase in biomass loading. Other researchers also noticed a similar result when they substituted modified horsetail for natural rubber as filler. An index to compare vulcanization numerically is provided by the cure rate index (CRI). The CRI increased as MB partially replaced CB, as Table 2 illustrates. At a 7.5 phr biomass concentration, the value increased to 10.15 from 7.96, which corresponded to NB-0 composites. The functional groups found on the algal biomass surface and its capacity to interact with the NBR matrix and CB are responsible for the increase in CRI. The decrease in CRI values for SBR composites may result from a greater interaction between fillers and escalated agglomeration of the biomass in the SBR composites.

#### Mechanical characterization

**Fig. 11:**
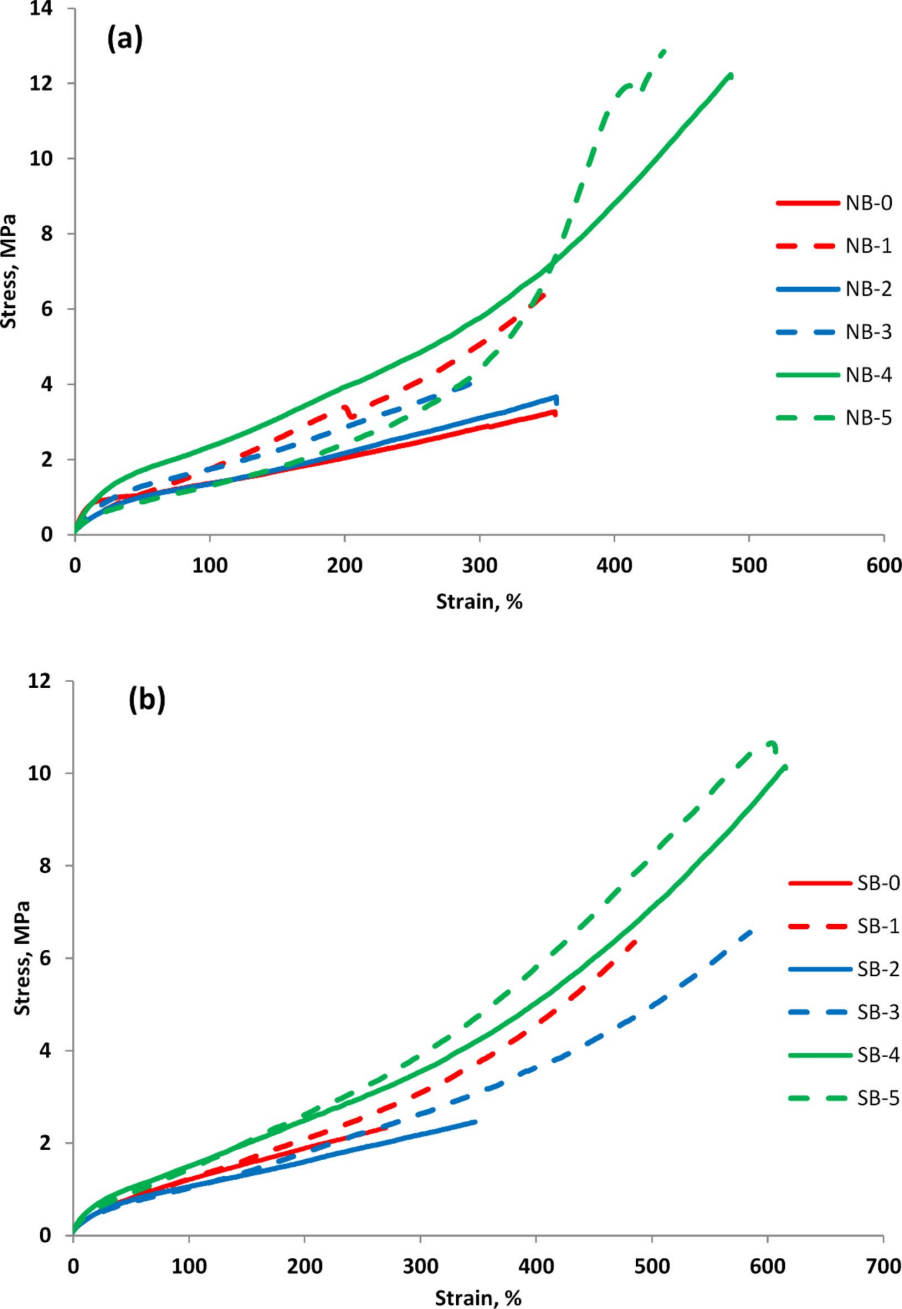
The tensile stress-strain curves of (**a**) NBR and (**b**) SBR vulcanizates filled with different MB/CB loading.

The effectiveness of biomass/CB fillers in strengthening the cured rubber is revealed by the tensile characteristics of the rubber composites. In comparison to gum vulcanizates of NBR and SBR, it is evident from Fig. 11 that both kinds of rubber-filled vulcanizates possess high strength. Additionally, the strength of the composites improves with an increase in biomass/CB loading. This may be explained by the fact that filled vulcanizates exhibit a better crosslinking density than gum vulcanizates, which improves as CB loading increases. This suggests that biomass and CB have the capacity to give filled vulcanizates more stiffness, which in turn decreases the mobility of the rubber chain.Similar results were also noted in earlier research^51^. The coupling agent (PEG) increases the contact between the biomass/CB and the polymeric chain, enhancing the matrix’s resistance to greater tensile stresses and strengthening the reinforcement^27^. Other researchers have confirmed that the application of coupling agents has a similar effect on the cross-linking kinetics of elastomeric materials^52^. It is noteworthy that, as Table 3 illustrates, all of the biomass/CB composites have more elongation at break than the gum vulcanizates of NBR and SBR. This indicates that the composites are more elastic due to the biomass/CB hybrid filler. The reason that the composites show improved elongation at break (Eb) may be attributed in part to the reinforcing impact of the MB nanoparticles that enhance the dispersion and interfacial adhesion inside the polymer matrix, boosting the elongation at break. Conversely, adding excessive biomass/CB could cause agglomeration or clustering that may cause stress concentration spots and restrict the mobility of the polymer chain. Because of the localized areas of weakness and decreased flexibility in the composite material, this clustering effect may lead to a reduction in elongation at break. Similar explanations were provided by other researchers^53^. Other researchers have confirmed that using biomass/CB has a comparable impact on the mechanical properties^54^.

**Table 3:** Mechanical properties.

Property	Samples code											
	NB-0	NB-1	NB-2	NB-3	NB-4	NB-5	SB-0	SB-1	SB-2	SB-3	SB-4	SB-5
Tensile strength (MPa)	3.26	6.4	3.66	6.79	12.2	12.85	2.33	6.34	2.45	6.56	10.15	10.65
Elongation at break (%)	354	345	354	446	486	418	267	478	344	577	615	598
100% Modulus (MPa)	1.37	1.76	1.35	1.81	2.4	2.47	1.21	1.23	1.01	1.24	1.5	1.69
200% Modulus (MPa)	2.05	2.1	2.19	2.94	3.9	4.33	1.75	2.11	1.64	1.93	2.5	2.8
Shore hardness (A)	46	56	51	54	51	55	47	52	49	54	59	57

It is evident from Table 3 that as compared to unfilled, both NBR and SBR composites, determined at 100% and 200%, and as biomass loading increases. This could be explained by the high biomass/CB loading, which reduces the distance between biomass/CB agglomerates and increases filler–filler interactions, which in turn enhances modulus at 100% and 200% elongation. Additionally, it is evident that NBR vulcanizates give a higher 100% and 200% modulus than SBR vulcanizates at a same biomass/CB loading, but their elongation at break is smaller^51^. This owes NBR-filled vulcanizates, which contain nitrile groups (polar groups), exhibit a significant crosslinking density and a good rubber-filler interaction, that enhances the stiffness and lowers the rigidity of the NBR vulcanizates,

A hardness test offers an efficient method to assess the changes in processing conditions or chemical affect mechanical characteristics. It is evident that when compared to unfilled vulcanizates, filled composites exhibit greater hardness. Additionally, the filled vulcanizate becomes harder than the gum vulcanizate as the biomass/CB amount increases due to increase the crosslinking density Table 3. The results above indicate that up to 50% of the CB filler can be successfully replaced with MB. Other investigations have also validated the beneficial impact of biomass use on the mechanical properties of rubber composites^19^.

Stress-strain behavior, which provides the first insights into the compatibility of filler (MB/CB) and rubber (NBR & SBR), is one of the important characteristics of elastomers. The stress-strain plots for NBR and SBR are clearly influenced by the size of agglomerates resulting from dual filler (MB/CB), the cross-linking density of the rubber matrix and the interactions between the rubber and filler. Fillers, curing agents, and the addition of compatibilizing agents can all be used to regulate these effects^55^. Figure 11 displays the stress-strain plots of NBR or SBR filled with various ratios of dual filler (15; 30; 45 and 60 phr) MB/CB, respectively. It is evident that the ratio of the two mixed fillers to one another and the addition of PEG acting as a interfacial modifier (coupling agent)^52^ had an impact on the initial slope of the stress-strain curves, whereas the incorporation of a large amount of MB/CB increased this initial slope. Because of the polarity difference between NBR and SBR as well as the interfacial interaction between MB and the rubber matrix, NBR and SBR composites have different mechanical properties.

This is because rubber (NBR & SBR) matrix and prepared fillers interact better now. Additionally, it is evident that increasing the investigated filler content in NBR or SBR rubber greatly improved stress. More rubber molecules are absorbed onto the dispersed MB/CB and take part in tensile orientation as a result of the introduction of MB/polyethylene glycol, which increases the compatibility between MB/CB and rubber (NBR &SBR). Furthermore, the addition of polyethylene glycol and MB may enhance the crosslinking density, which may positively impact the tensile strength. These curves were found to be sensitive to the concentration of MB/CB. Comparatively, vulcanizates loaded with 45 phr of biomass/CB showed greater values of failure stress (NB_**4**_ & SB_**4**_), followed by that containing 30 phr of MB (NB_**5**_ & SB_**5**_) and then those loaded with other concentrations of MB such as (15 phr, NB_**3**_ or SB_**3**_). Additionally, it was observed that the composites’ stiffness increased as the biomass/CB loading increased. Otherwise, NBR or SBR loaded with MB/CB exhibited normal elastomer behavior, which can be split into two stages: The first stage of minor strain, in which the entangled chains relaxed as the stress gradually, grew. The second stage of medium strain, where the elongated chains’ high stretching caused the tension to build at a regular rate, was followed by a high stretching stage. Incorporating MB into NBR vulcanizates with coupling agents also contributes to the establishment of filler-rubber interaction, which creates more physical crosslinks in the vulcanizates’ network structure^54^. The energy absorbed can therefore be expressed as follows: As a result, it is anticipated that the energy absorbed per unit volume (ea.) in the deformed rubber vulcanizates will rise.

3 $${\mathbf{Ea}}.=\oint {\mathbf{\varsigma }}\left( {\mathbf{\zeta }} \right){\text{ }}{\mathbf{d\zeta }}$$

where ς represents stress as a function of strain; thus, a smaller region under the stress-strain curve indicates a lower capacity for energy absorption. The NBR composites containing MB/CB (7.5/7.5 phr) has the lowest energy absorption as a result of having the smallest area under the curve. These findings are consistent with those shown in Tables 5 and 6, which indicates that the addition of 7.5 phr of MB to the rubber matrix resulted in a decrease in strain energy. Conversely, the highest energy absorption is seen in the vulcanizates NBR or SBR, which contain (22.5 & 30 phr) of MB and CB. These results reveal a decrease in strain energy due to the integration of MB in rubber vulcaniztes, which is in good agreement with the data shown in Table 4. Additionally, Table 4’s results revealed that, the value of strain energy for rubber/CB is higher than rubber/MB/CB. Regarding energy consumption, CB is more wasteful than MB. The manufacture and disposal of rubber compounds have the potential to have a very positive environmental impact, which might be mitigated by using M as filler. According to earlier research, strain energy was decreased in rubber composites when MB partially replaced CB^19^.

**Table 4:** The value of constant C1 and C2 for different concentrations of microalgal biomass/CB.

Properties/no of sample	NB1	NB2	NB3	NB4	NB5	SB1	SB2	SB3	SB4	SB5
Strain energy, MJ	387	278	279	1111	778	303	119	341	740	786
C1, MPa	0.01137	0.01039	0.01079	0.02571	0.01612	0.01034	0.01024	0.008798	0.01464	0.009017
C2, MPa	0.06104	0.06008	0.0643	0.06689	0.07115	0.0599	0.0545	0.060022	0.06942	0.06822

To evaluate the strength of the interfacial relationship between the rubber matrix and filler in more detail, utilize the Mooney–Rivlin equation^56,57^. In other words, the Mooney–Rivlin equation is used to assess the tensile behaviors of rubber composites in order to uncover the network structure characteristics and the reinforcing mechanism of MB/CB towards examined rubber (NBR & SBR):

4 $$\sigma ~red=~\frac{\sigma }{{\varPsi - ~\frac{1}{{{\varPsi ^2}}}}}~=2{C_1}+2\frac{{{C_2}}}{\varPsi }$$

**Fig. 12:**
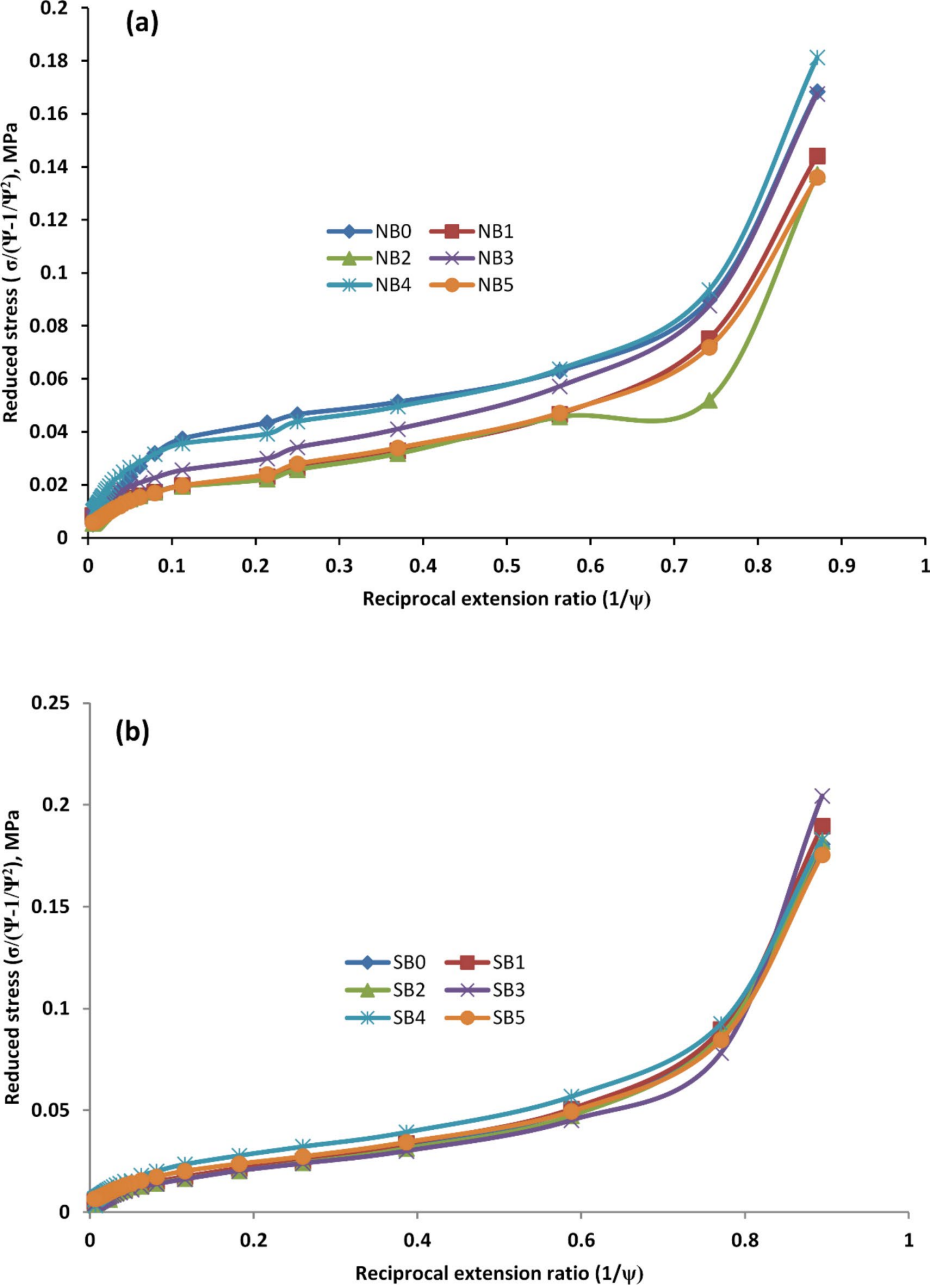
Moony-Rivlin plots for (**a**) NBR and (**b**) SBR vulcanizates filled with different MB/CB loading.

The engineering stress is represented by σ red, the decreased stress; ${\Psi - ~\frac{1}{{{\Psi ^2}}}}$ suggested by the molecular theory of rubber elasticity, the nominal stress is represented by σ; the strain ratio is represented by Ψ; the constants C_**1**_ and C_**2**_ are independent of Ψ; and the applied stress has been divided by the strain function. The crosslink density of the composites is expressed by the value of C_**1**_, while the quantity of physical and additional unstable crosslinks, such as plaster/plaster contact, entanglement, and filler/rubber interactions, is related to C_**2**_. The Mooney-Rivlin plots of the rubber composites under evaluation are shown in Fig. 12a and b. The interface strength between the studied rubber matrix and biomass/CB can be determined by measuring the upturn point $\varPsi _{{Up}}^{{ - 1}}~$. A larger upturn $\varPsi _{{Up}}^{{ - 1}}~$ value indicates a more interaction between the rubber matrix and MB/CB, which is advantageous for improving the dispersion of filler. Figure 12 shows that the upturn $\varPsi _{{Up}}^{{ - 1}}~$ values increased with increasing MB/CB loading for both NBR and SBR composites. This suggests that good dispersion of MB/CB in rubber matrix and strong interaction between them. It is notable that, in accordance with the SEM results, the dispersion of biomass/CB in NBR vulcanizates is superior to that of MB/CB in SBR vulcanizates, as evidenced by an increase in upturn $\varPsi _{{Up}}^{{ - 1}}~$ value.

It is evident that adding MB in different ratios to partially replace CB in the NBR and SBR composites increased the C_**2**_ value. On the other hand, the Mooney-Rivlin constant C_**2**_ value, which is related to the intermolecular forces and flexibility between rubber chains, increases as the amount of MB increases^58,59^. NBR/MB/CB are shown to have greater C_**2**_ values among the composite components, indicating the presence of higher chain entanglement, which is indicative of improved molecular level mixing. As a result, the Mooney-Rivlin equation and the observed mechanical property changes can be directly correlated. Other researchers have also confirmed the beneficial impact of using MB on the stress-strain of rubber composites^19^.

#### Thermodynamic features

**Fig. 13:**
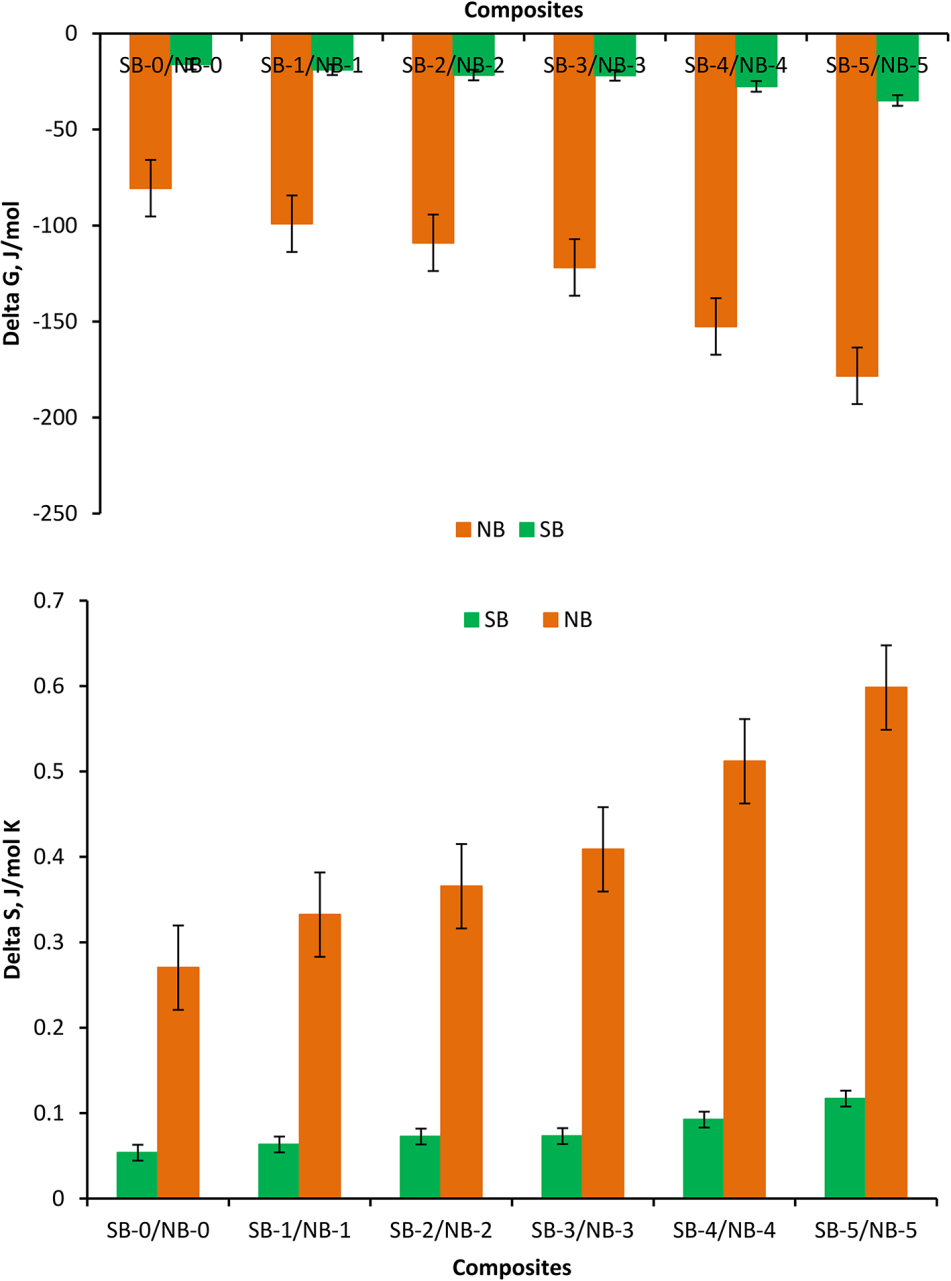
The thermodynamics parameters ΔG and ΔS for NBR and SBR composites.

Figure 13 illustrates the thermodynamics parameters ΔG and ΔS for NBR and SBR composites containing same amount of MB/CB. Really, the argument of thermodynamic parameters of NBR and SBR composites is employed to study the diffusion of MB/CB in NBR or SBR matrix. These graphs clearly show that the greater negative shift in free energy values (ΔG), the larger the compatibility between the composites. When ΔG < 0, a thermodynamically stable system is generated. ΔG values in the present search decrease as the MB/CB filler content rises. Because the MB/CB filler impacts the reduction in dispersed phase size, the interfacial region increases to a specific level when the filler loading increases. The ΔG values produce as the MB/CB content increases. The value of ΔG indicates that NBR/ (30/30) MB/CB demonstrates more flexible behavior compared to SBR/ (30/30) MB/CB as ΔG is closely associated with the material’s elastic properties^60^. The better elastic behavior of NBR/ (30/30) MB/CB is described by providing higher degree of interaction between NBR matrix and MB/CB is higher than SBR matrix and MB/CB. The data represented in Fig. 13 depicts the conformational entropy (ΔS) of NBR/ (30/30) MB/CB is greater as compared to SBR/ (30/30) MB/CB. The probable explanation may be due to uniform dispersion of MB/CB inside the NBR rubber matrix is responsible for the upper value of ΔS in NBR / (30/30) MB/CB than other content or SBR loaded with investigated fillers the same result confirmed by Abdelsalam et al.^53^.

#### Swelling properties

The swelling characteristic yields details on the percentage of soluble fraction (the fraction of chains that are not part of the network) and the density of the resulting network. Fillers can minimize swelling to some extent through interactions between the filler and filler, but they can also act as extra cross-links due to interactions between the filler and rubber. It is clear that the values of EQ_**filler**_/EQ_**rubber**_ decreased when MB was added to the NBR and SBR composites in different ratios to partially replace CB. The values (1/EQ_**m**_) can be utilized to calculate the effect of the interaction between the filler under study MB/CB and NBR or SBR. Reduced interaction between the rubber matrix and the investigated filler was the result of higher values of (EQ_**filler**_/EQ_**rubber**_). The data presented in Tables 5 and 6 indicates that when NBR/ (30/30) MB/CB was used, the ratio of the studied filler to NBR rubber was greater than that of SBR/(30/30 MB/CB. This indicates that the smallest value of (EQ_**filler**_/EQ_**rubber**_) and the largest value of (1/EQ_**m**_) were obtained in this scenario. These outcomes are the consequence of crosslink production, which was identified by the molecular weight between crosslinks (Mol.c) based on equilibrium swelling measurements. It is also important to note that the soluble fraction values of NBR rubber filled with various content of CB and MB are higher than those of SBR rubber, CB, and MB or unfilled rubber vulcanizates. This is because CB and MB are partially soluble in organic solvents, which is why they are used as filler in NBR rubber^61^. Conversely, when the concentrations of MB were increased until 30 phr, the soluble fractions dropped. The observed rise in the concentration of MB may have been caused by their hindering effect on the vulcanization process of the NBR rubber compositions under investigation. The same pattern may be seen in the parameters of the SBR with varying amounts of MB. The data above indicate that 30 phr was the ideal concentration of MB, which demonstrated promising qualities.

**Table 5:** Swelling characteristics for NBR vulcaniztes with different levels of microalgal biomass/carbon black content.

Sample numbers	EQm, %	Soluble fraction, %	Vol. fract	ν_den_, (mol/cm^3^)	Mol.C (g mol^-1^ )	$\frac{{{\text{E}}{{\text{Q}}_{{\text{filler}}}}}}{{{\text{E}}{{\text{Q}}_{{\text{NBR}}}}}}$	$\frac{1}{{{\text{E}}{{\text{Q}}_{\text{m}}}}}$
NB-0	187.3	7.7	0.3481	2.4685 × 10^−4^	2025.518	—	0.5339
NB-1	167.09	7.3	0.3744	3.0123 × 10^−4^	1659.862	0.8921	0.5985
NB-2	158.37	7.225	0.3870	3.3044 × 10^−4^	1513.120	0.8455	0.6314
NB-3	148.72	5.865	0.4021	3.6801 × 10^−4^	1358.668	0.7940	0.6724
NB-4	130.58	4.77	0.4337	4.5830 × 10^−4^	1090.995	0.6972	0.7658
NB-5	119.04	4.29	0.4565	5.3411 × 10^−4^	936.1423	0.6356	0.8936

**Table 6:** Swelling characteristics for SBR vulcaniztes with different levels of microalgal biomass/carbon black content.

Sample numbers	EQm,%	Soluble fraction, %	Vol. fract	ν_den_, (mol/cm^3^)	Mol.C (g mol^-1^ )	$\frac{{{\text{E}}{{\text{Q}}_{{\text{filler}}}}}}{{{\text{E}}{{\text{Q}}_{{\text{SBR}}}}}}$	$\frac{1}{{{\text{E}}{{\text{Q}}_{\text{m}}}}}$
SB-0	365	7.53	0.2151	6.54011 × 10^−5^	7645.134	—	0.2740
SB-1	338	10.02	0.2283	7.62532 × 10^−5^	6557.099	0.9260	0.2959
SB-2	317	9.04	0.2398	8.66449 × 10^−5^	5770.681	0.8685	0.3155
SB-3	315.73	7.91	0.2405	8.73394 × 10^−5^	5724.795	0.8650	0.3167
SB-4	282.71	7.17	0.2613	10.8724 × 10^−5^	4598.805	0.7745	0.3537
SB-5	252.34	6.64	0.2838	13.5976 × 10^−5^	3677.110	0.6913	0.3963

Tables 5 and 6 illustrates the variations of the crosslink density for SBR and NBR with different MB and CB loading. As expected, when more and more of the CB is gradually replaced by MB loading, the crosslink density in rubber composites rises. These crosslinks limit the rubber chains’ ability to stretch in response to swelling and increase the difficulty of oil diffusing into the spaces between rubber molecules, thereby reducing the percentage of swelling^62^. As was previously established, metal oxides in MB are the reason of the elevated state of cure. Higher crosslink density is reported with NBR than with SBR at the same MB input. It could be referring to the quantity of double bonds or active sites that are present in the chains of the various elastomers (NBR and SBR). In the chemical structure of each elastomer, for instance, one double bond can be found for every seven carbons in NBR and one for every twelve carbons in SBR^63^.

MB’s tortuosity and polarity prevent non-polar solvent molecules from penetrating the NBR matrix. The network structure of the studied fillers (MB/CB) created through hydrogen bonding function as physical crosslinks or entanglements for elastomers^64^. The reason NBR/MB/CB has the highest crosslink density compared to SBR/MB/CB is because of the material’s decreased free volume availability, which results from the high interfacial adhesion between the NBR rubber matrix and MB/CB. These findings are confirmed through study of crosslink density^64^.

### Conclusions

Conventional petroleum-based CB fillers in rubber composites face several issues, including environmental concerns over their non-biodegradability and the rising costs of raw materials. This has prompted the search for sustainable alternatives. The current study investigated the use of MB as a partial replacement for CB in NBR and SBR rubber composites.

The key findings are as follows:

- SEM-EDX analysis showed the MB has distinct shapes and smooth surfaces, with accumulation of carbon, oxygen, and nitrogen on the surface. SEM observations revealed good adhesion and dispersion of the MB/CB within the rubber matrix.
- The inclusion of MB decreased the cure rate of the composites, likely due to partial obstruction of inter-chain bond formation and creation of additional crosslinks.
- The MB-filled vulcanizates exhibited higher tensile strength and elongation at break compared to the unfilled samples, indicating improved elasticity.
- The increased crosslink density suggests strong interactions between the fillers and the rubber matrix.

In conclusion, the study demonstrated that MB can effectively replace up to 50% of the carbon black filler, while maintaining satisfactory mechanical properties. This replacement can significantly reduce the use of the carcinogenic carbon black component and lower the overall cost of these rubber compounds for tire tread applications.

NBR/MB/CB composites could be utilized in the manufacturing of well-known rubber goods, such as motor mounts, floor heating, automobile radiator and brake hoses, and vehicle radiator hoses. These composites are the industry standard for use in the oil and gas and automotive sectors due to their superior nonpolar solvent and oil resistance, as well as their excellent mechanical properties.

Applications for substituting some of the carbon black (CB) in SBR composites with microalgal biomass include packing cushion foams, floor vehicle tires, adhesives, and footwear.

## Electronic supplementary material

Below is the link to the electronic supplementary material.

Supplementary Material 1

## Data Availability

Data will be made available on request from the corresponding author email: doaa.samiir@yahoo.com.
