# Supplementary material for: Advancements in microalgal biomass conversion for rubber composite applications
Source: Sci Rep. 2025 Jan 4;15:810. doi: 10.1038/s41598-024-82878-7 (PMC11700149; doi:10.1038/s41598-024-82878-7)
Supplement: Supplementary file 1 — Supplementary Material 1 [file 41598_2024_82878_MOESM1_ESM.pdf]

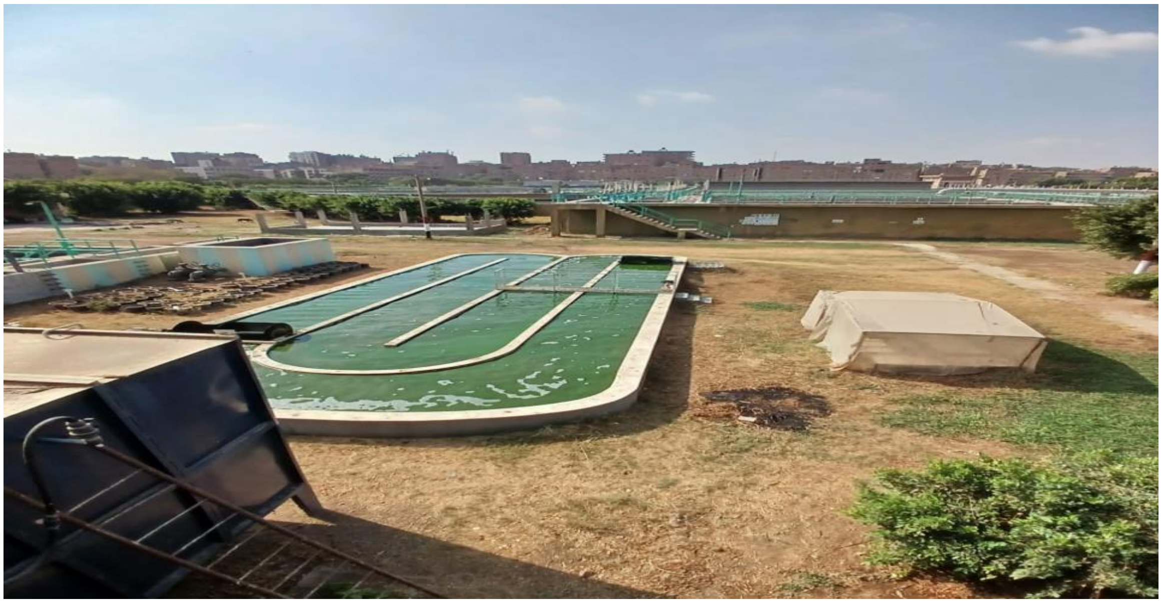

S1 High rate algal pond (HRAP) constructed at Zenin wastewater treatment plant, Giza, Egypt.

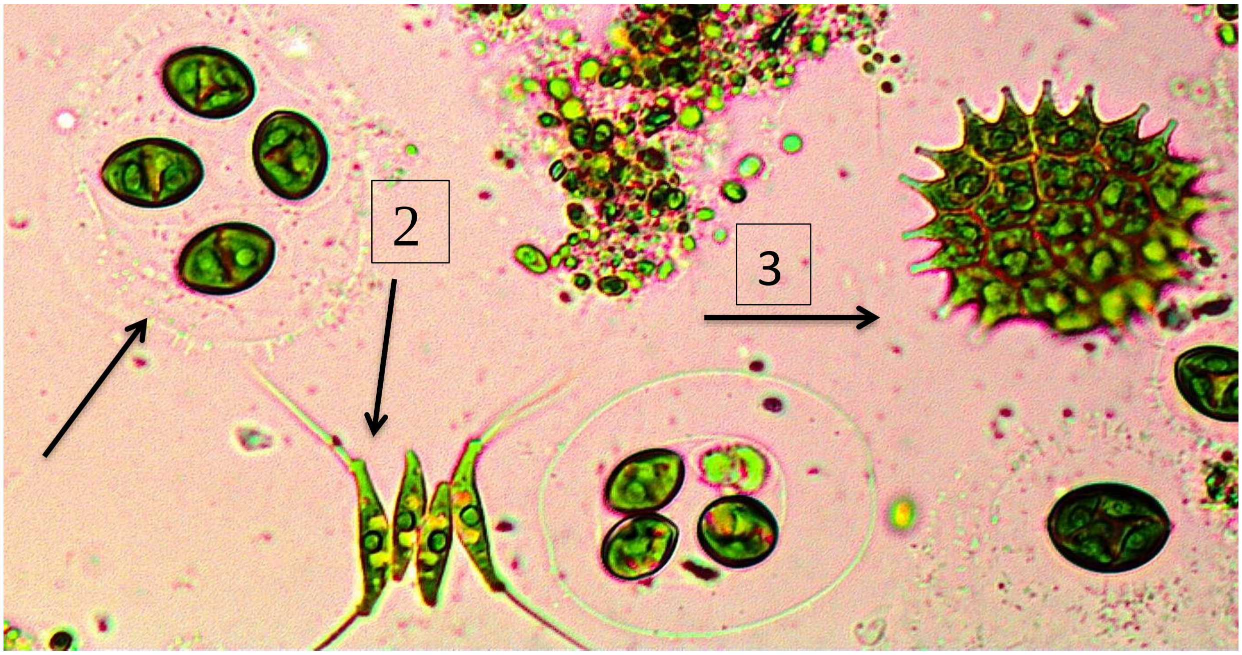

S2 Microalgal species detected in HRAP where, (1) *Oocystis parva ;* **(2)** *Scenedesmus quadricauda;* **(3)** *Pediastrum gracillimum*
